# Non-coding RNA *LINC00473* mediates decidualization of human endometrial stromal cells in response to cAMP signaling

**DOI:** 10.1038/srep22744

**Published:** 2016-03-07

**Authors:** Xiao-Huan Liang, Wen-Bo Deng, Yue-Fang Liu, Yu-Xiang Liang, Zong-Min Fan, Xiao-Wei Gu, Ji-Long Liu, Ai-Guo Sha, Hong-Lu Diao, Zeng-Ming Yang

**Affiliations:** 1 College of Veterinary Medicine, South China Agricultural University, Guangzhou, China; 2 Department of Biology, Shantou University, Shantou, China; 3 Reproductive Medicine Center, Bailu Hospital, Xiamen, China; 4 Reproductive Medicine Center, Renmin Hospital, Hubei University of Medicine, Shiyan, Hubei, China

## Abstract

Decidualization is an essential step in the establishment of pregnancy. However, the functional contributions of long intergenic noncoding RNAs (LincRNAs) to decidualization have not been explored. To explore the regulation and role of LincRNAs during human decidualization, human endometrial stromal cells (HESCs) are induced to undergo *in vitro* decidualization by treating with estradiol-17β, db-cAMP and medroxyprogesterone acetate. *LINC00473 (LINC473)* expression is highly induced in HESCs after decidual stimulus. We found that cAMP-PKA pathway regulates the expression of *LINC473* through IL-11-mediated STAT3 phosphorylation. RNA interference-mediated down-regulation of *LINC473* inhibits *in vitro* decidualization. These results suggested that *LINC473* might be functionally required for human decidualization. This is the first report demonstrating the presence of LincRNA during human decidualization.

Decidualization, the transformation of endometrial stromal cells into specialized secretory decidual cells, is important for pregnancy by restraining trophoblast invasion and ensuring adequate homeostasis. Both excessive and inadequate invasion may lead to disastrous consequences^1^. Although protein-coding genes have been extensively characterized during human decidualization, whether long intergenic non-coding RNA (LincRNA) has a role in decidualization has not been studied. LincRNAs are more and more recognized as active molecules instead of “transcriptional noise”. Studies have revealed the involvements of LincRNAs in regulation of multiple biological processes^2^. However, the LincRNAs that participate in decidualization remain unclear.

The convergence of cAMP and progesterone signaling pathways is important for human decidualization^3^. cAMP sensitizes human endometrial stromal cells (HESCs) to progesterone via activation of the protein kinase A (PKA) signaling^3^. In human ciliary smooth muscle cells, long intergenic non-coding RNA 473 (*LINC473*) expression is obviously induced upon activation of the cAMP signaling pathway^4^. Because cAMP is a critical mediator of human decidualization, we hypothesized that cAMP-induced *LINC473* expression might be important for human decidualization. However, the regulation and functional role of *LINC473* during human decidualization remain unknown.

As a major mediator of IL-6 family signaling, STAT3 is important for mouse embryo implantation^5^,^6^,^7^. In humans, inhibition of STAT3 expression will impair decidualization^5^,^8^. However, the precise mechanism underlying the regulation of STAT3 on stromal decidualization remains unknown.

In this study, we identified a cAMP-induced expression pattern of *LINC473* in the progress of decidualization. The cAMP-activated STAT3 plays a critical role in the induction of *LINC473*. We further provided evidence that knockdown of *LINC473* attenuates the expressions of decidual markers prolactin (PRL) and insulin-like growth factor binding protein 1 (IGFBP1).

## Results

### Regulation of *LINC473* expression by cAMP

**Figure 1:**
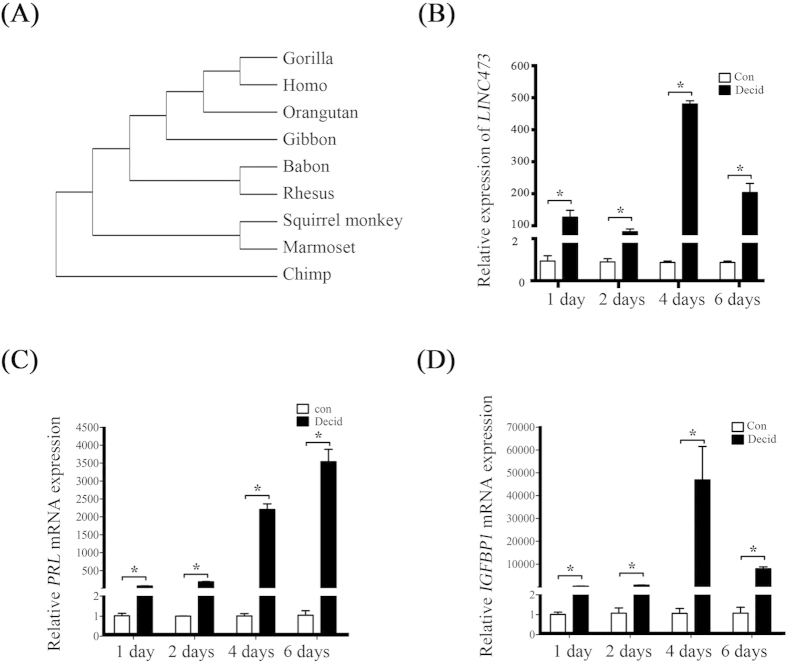
*LINC473* is induced during decidualization. (**A**) Simplified hylogenetic tree showing the evolutionary characteristics of *LINC473* in primates. (**B**) *LINC473* expression increases at different time points after decidualization. (**C**) *PRL* expression during decidualization. (**D**) The expression of *IGFBP1* during decidualization. Each treatment was performed with at least three biological replicates. Error bars represent standard errors. ^*^P < 0.05.

As a remarkable property of LincRNAs is their low conservation^9^, we first did the evolutionary characteristic assay of *LINC473*. Based on our evolutionary characteristic analysis, *LINC473* was identified as a primate-specific LincRNA (Fig. 1A).

Under *in vitro* decidualization, real-time PCR showed a strong induction of *LINC473* after 1, 2, 4 and 6 days of decidualization, respectively (Fig. 1B). IGFBP1 and PRL, two decidual markers, were also strongly expressed (Fig. 1C,D). These results indicated that decidual stimulus was sufficient to induce and sustain *LINC473* expression.

**Figure 2:**
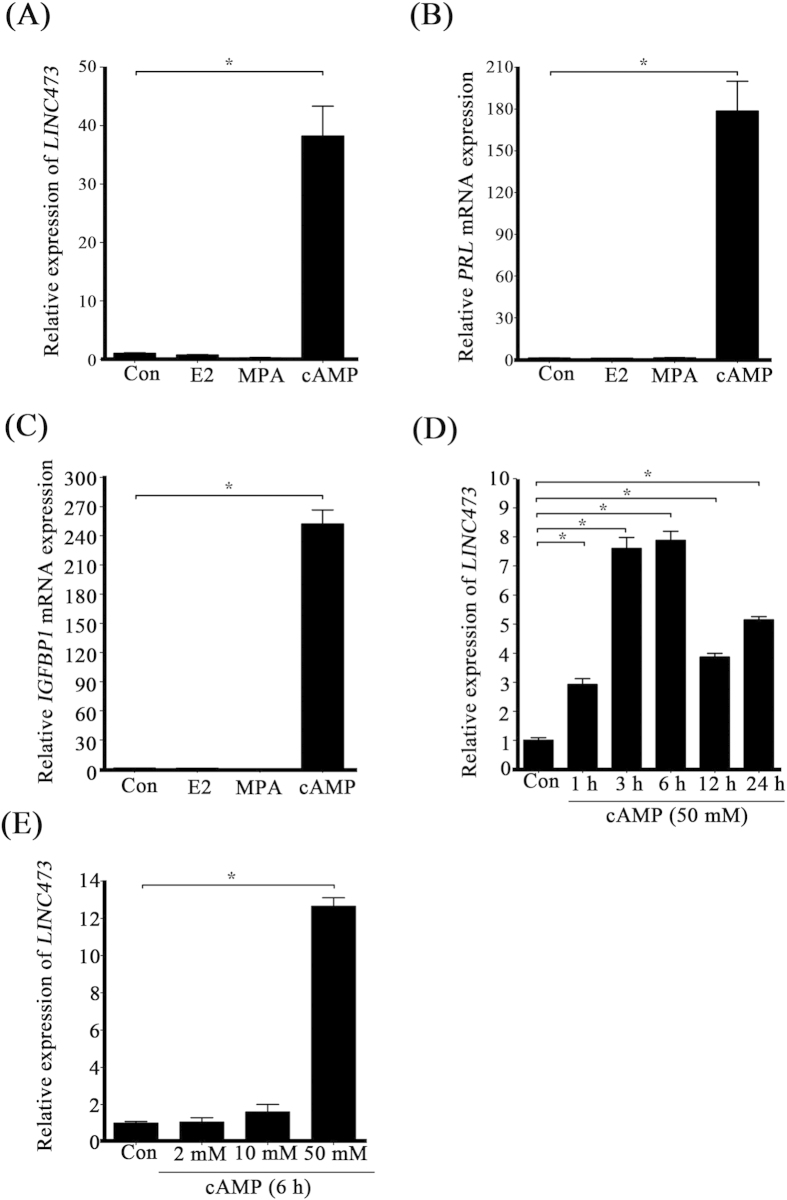
*LINC473* induction is under the control of cAMP. (**A**) Relative expression level of *LINC473* after stromal cells were treated with E2, P4 or cAMP for 24 h. (**B**) Effects of E2, P4 or cAMP on *PRL* expression. (**C**) Effects of E2, P4 or cAMP on *IGFBP1* expression. (**D**) The time courses of cAMP-induced *LINC473* expression. (**E**) Effects of cAMP of different concentrations on *LINC473* expression. Each treatment was performed with at least three biological replicates. Error bars represent standard errors. ^*^P < 0.05.

In our study, *in vitro* decidualization was induced with cAMP, progesterone and estrogen. To examine how one of these distinct factors regulates *LINC473*, HESCs were treated with cAMP, progesterone or estrogen, respectively. Real-time PCR analysis demonstrated that *LINC473* expression increased dramatically upon cAMP treatment, but not by progesterone or estrogen treatment (Fig. 2A). The transcriptional levels of IGFBP1 and PRL, were also up-regulated by cAMP significantly (Fig. 2B,C). We then examined effects of time and dosage courses of cAMP on *LINC473* expression. The cAMP regulation on *LINC473* was rapid and dosage-dependent (Fig. 2D,E). Together, these results showed that the induction of *LINC473* during decidualization was under the control of cAMP.

### *LINC473* regulation by cAMP via induction of STAT3

**Figure 3:**
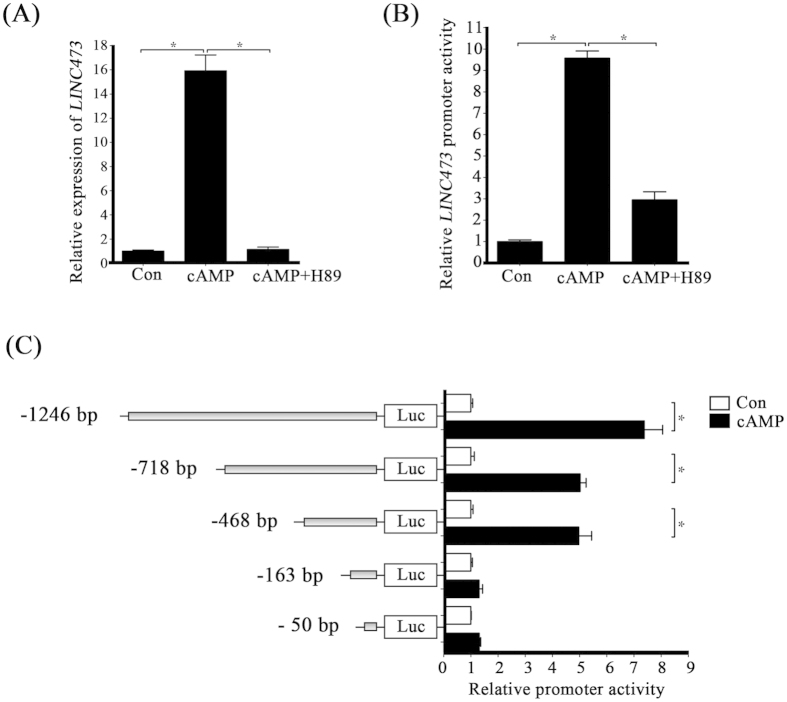
cAMP regulates *LINC473* via H89. (**A**) HESCs were treated with cAMP for 6 h. H89 prevents the cAMP-induced increase of *LINC473* expression level. (**B**) HESCs transfected with LINC473 promoter reporter plasmid were treated with cAMP for 24 h. The promoter activity of *LINC473* is decreased with H89 treatment. (**C**) Luciferase activity of *LINC473*-promoter reporters with different lengths after HESCs were treated with cAMP for 24 h. Each treatment was performed with at least three biological replicates. Error bars represent standard errors. ^*^P < 0.05.

To further confirm the cAMP regulation on *LINC473* transcription, *LINC473* promoter reporter was constructed for luciferase analysis. Because cAMP signaling is transduced exclusively by PKA^10^, H89, a selective PKA signaling inhibitor, was used to investigate whether PKA signaling pathway is involved in the induction of *LINC473*. The cAMP inductions on *LINC473* expression and promoter activity were both attenuated by H89 (Fig. 3A, B). Together, these results showed that the induction of *LINC473* during decidualization is under the control of cAMP-PKA pathway.

To identify the functional promoter region regulated by cAMP, different lengths of promoter truncations were constructed. The luciferase activities of −1246, −718 and −468 bp promoter reporters increased remarkably after cAMP treatment (Fig. 3C). However, there’s no obvious induction on luciferase activity for the −163 and −50 bp fragments. These results indicated that the importance of the −163 bp to −468 bp region to the effects of cAMP.

### STAT3 activation involves induction of IL-11 during the early stage of decidualization

**Figure 4:**
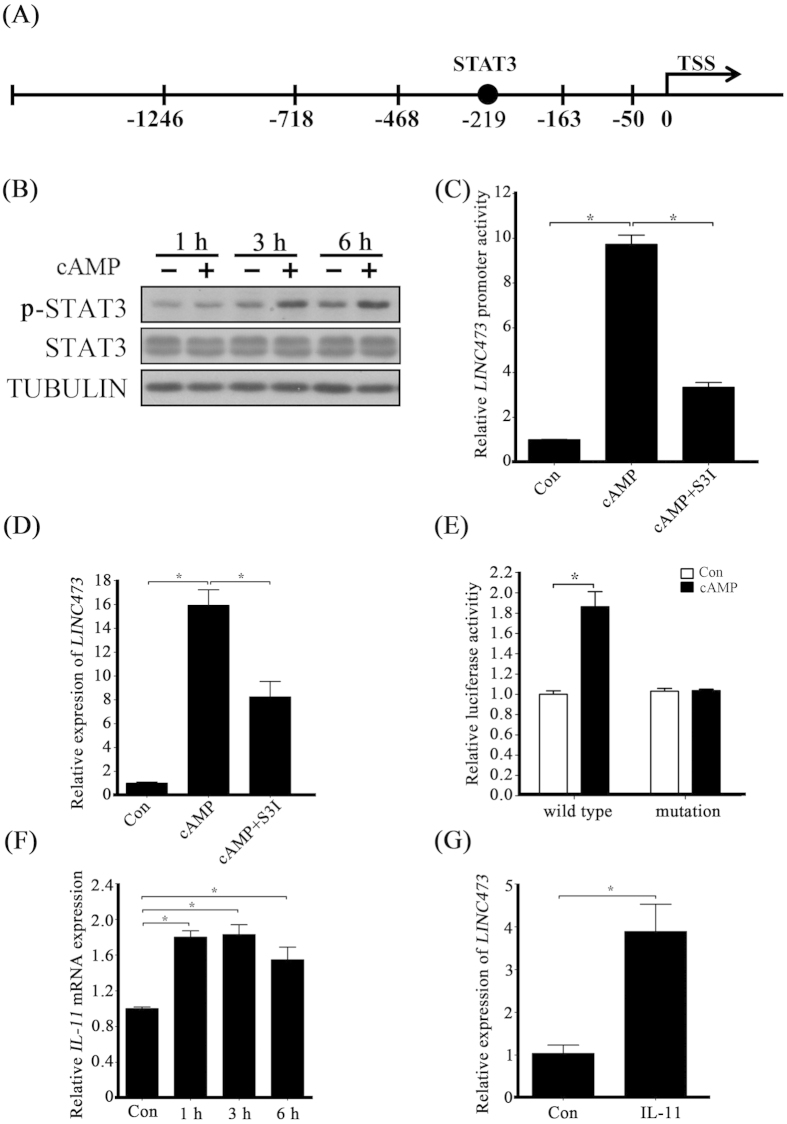
cAMP regulation on *LINC473* via STAT3. (**A**) Schematic representation of *LINC473* promoter region showing the location of STAT3 binding. (**B**) cAMP induces the phosphorylation of STAT3 protein. (**C**) Effects of STAT3 inhibitor S3I on *LINC473* promoter activity when HESCs were treated with cAMP. (**D**) Effects of STAT3 inhibitor on cAMP-induced *LINC473* expression. (**E**) Mutation of STAT3 binding site attenuated the cAMP induction on *LINC473* promoter activity. (**F**) Stimulation of IL-11 mRNA expression by cAMP. (**G**) *LINC473* expression is induced by IL-11 treatment in HESCs. Each treatment was performed with at least three biological replicates. Error bars represent standard errors. ^*^P < 0.05.

Evidences have showed that non-coding RNAs are directly regulated by transcription factors^11^. Therefore, we explored whether *LINC473* is controlled by known decidualization-related transcription factors. Based on our *in silico* analysis of the −163 to −468 bp region relative to the *LINC473* transcription start site (TSS), there was one binding site for STAT3 in this region (Fig. 4A). Because STAT3 is essential for human decidualization^8^, we assumed that STAT3 may be involved in the regulation of *LINC473*. We first monitored STAT3 expression upon cAMP treatment. The level of STAT3 mRNA showed no change in the early stage of cAMP treatment (data not shown). However, the level of phosphorylated STAT3 protein was upregulated by cAMP administration (Fig. 4B). To determine whether STAT3 is required for *LINC473* induction by cAMP, S3I-201 (S3I), a selective inhibitor of STAT3 phosphorylation, was used to treat cells at the presence of cAMP. The assay on *LINC473* promoter activity showed that S3I administration weakened the induction of *LINC473* promoter activity by cAMP (Fig. 4C). The real-time PCR results displayed a similar pattern (Fig. 4D). To further confirm the function of STAT3, we mutated the STAT3 binding site and found that the mutation of STAT3 binding site attenuated the cAMP effects on *LINC473* promoter reporter activity (Fig. 4E). These results indicated that STAT3 is required for cAMP regulation on *LINC473*.

To explore the molecular pathways through which STAT3 is activated, we searched the microarray data from cAMP-treated HESCs^12^. Based on their analysis, the expression level of IL-11, an IL-6 family member, was rapidly induced by cAMP. Our real-time PCR results also confirmed the induction of IL-11 by cAMP in HESCs (Fig. 4F). *LINC473* mRNA expression was upregulated by IL-11 administration (Fig. 4G). These findings demonstrate that the phosphorylation of STAT3 may be involved in cAMP induction of *LINC473*.

### PGE2 regulation on *LINC473* expression

As cAMP production stimulated by PGE2 is important for decidualization, we examined whether PGE2 can regulate *LINC473* expression. We performed dosage and time course experiments of PGE2 treatment on *LINC473* expression. Real-time PCR results showed that PGE2 upregulated *LINC473* expression (Fig. S1 A,B). The expression of IL-11 was also up-regulated by PGE2 (Fig. S1C). Western blot showed that the level of phosphorylated STAT3 protein was increased upon PGE2 treatment (Fig. S1D). PGE2 regulation on *LINC473* was attenuated by H89 (Fig. S1E).

### Functions of *LINC473* during decidualization

**Figure 5:**
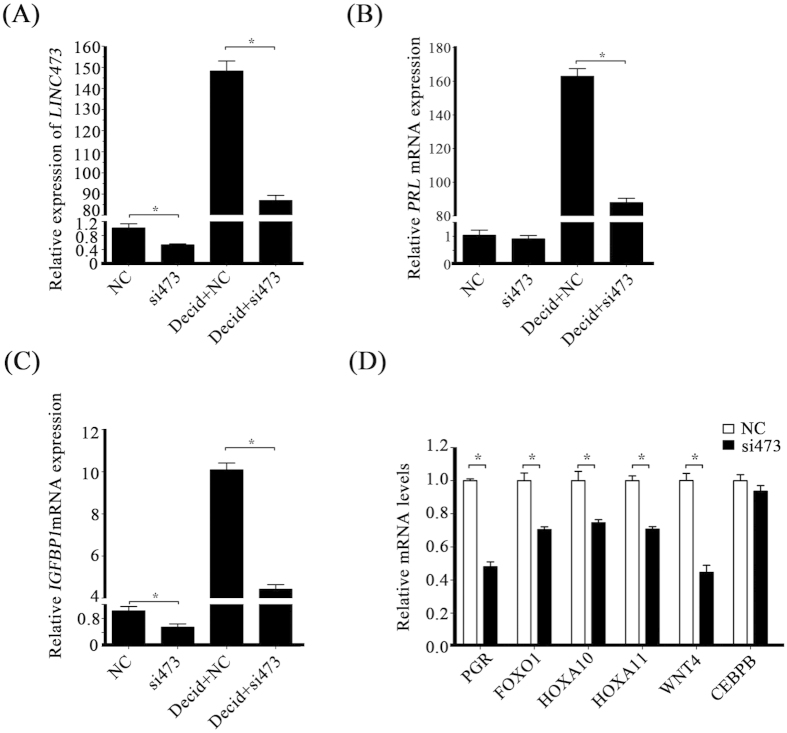
Differentially expressed decidualization-related genes in HESCS transfected with *LINC473* siRNA. (**A**) *LINC473* expression after silencing with siRNA. (**B**) Knockdown of *LINC473* decreases *PRL* expression in decidualized HESCs. (**C**) Knockdown of *LINC473* decreases *IGFBP1* expression in decidualized HESCs. (**D**) Effects of *LINC473* siRNA on the expression of decidualization-related genes. The expression levels of PGR, FOXO1, HOXA10, HOXA11, WNT4, and CEBPB were decreased by *LINC473* knockdown. Each treatment was performed with at least three biological replicates. Error bars represent standard errors. ^*^P < 0.05.

To analyze the function of *LINC473*, *LINC473* was knocked down by siRNA under *in vitro* decidualization. Real-time PCR showed that transfection of HESCs with *LINC473* siRNA significantly reduced *LINC473* expression (Fig. 5A). *LINC473* knockdown also led to significant reductions in the mRNA levels of both PRL and IGFBP1 in decidualized HESCs (Fig. 5B,C). These results revealed an essential role of *LINC473* in human decidualization. Additionally, the expressions PGR, FOXO1, HOXA10, HOXA11, and WNT4 were down-regulated upon *LINC473* knockdown (Fig. 5D). These results indicated the importance of *LINC473* during decidualization.

## Discussion

In contrast to the well-studied microRNAs, LincRNAs act through diverse mechanisms, as both positive and negative regulators of gene expression. Thousands of LincRNAs have been annotated in humans^13^, but the functions on the great majority of them remain unclear. A remarkable property of LincRNAs is their low interspecies conservation. Up to date, there are approximately 11,000 primate-specific LincRNAs and 2,500 conserved LincRNAs^9^. Our findings demonstrated that primate-specific *LINC473* might play a critical role in human decidualization.

Several long non-coding RNAs have been reported in human uterus, including HOXA11 antisense^14^, SRA gene^15^, FGF-AS^16^, and EMX2OS^17^. LincRNA HOTAIR is reported to be important in the endometrial carcinogenesis^18^. However, the functional role of LincRNAs during the process of decidualization has not been reported.

There is overwhelming evidence that initiation of the decidual process requires cAMP signaling^3^. When HESCs were treated with progesterone, estrogen or cAMP, a significant *LINC473* induction by cAMP was observed, but not by progesterone or estrogen, suggesting that cAMP might be the key inducing factor of *LINC473* under human *in vitro* decidualization. PKA is the principle intracellular target for cAMP in mammalian cells^19^. In this study, PKA inhibitor H89 prevented the cAMP-dependent enhancement of *LINC473*. It has been reported that cAMP can induce human stromal cells to secrete IL-11 as decidualization progresses^20^. We showed that treatment of IL-11 can increase the *LINC473* expression, suggesting that cAMP, at least partially, stimulates the expression of *LINC473* via an IL-11-dependent pathway. As PGE2 has been considered as a physiological generator of cAMP^21^, we examined whether PGE2 can regulate *LINC473* expression. The expression and secretion of IL-11 can be induced by PGE2 in HESCs^20^. It is possible that PGE2-stimulated cAMP induces the expression of IL-11, which drives the induction of *LINC473*.

Previous studies have identified LincRNAs that are directly regulated by transcription factors^22^. Transcription factor STAT3 has been identified as an critical regulator during human decidualization^8^. Our promoter analysis indicated that the cAMP-sensitive promoter region of *LINC473* located from –163 to –468 bp contains STAT3 binding site. Here we report a unique finding that, besides protein-coding gene, STAT3 regulates the decidualization progress through *LINC473*.

LincRNAs could work either in cis or in trans. Their roles in regulating gene expression could be negative or positive^23^. To study the mechanism of *LINC473* regulation, we first assumed the in cis regulation. Phosphodiesterase 10 (PDE10) gene, coding an enzyme hydrolyzing cAMP^24^, is the nearest gene in the *LINC473*-localized chromatin. However, no change of PDE10 expression was observed with *LINC473* knockdown (data not shown), suggesting that no relationship exists between these two molecules.

In this study, we provided additional evidence of *LINC473* regulation on several crucial decidual transcription factors and WNT4. Our results showed that *LINC473* knockdown leads to a marked down-regulation of FOXO1, progesterone receptor (PGR), HOXA10, HOXA11 and WNT4 at the transcriptional level, but did not significantly affect the expression of CEBPB. Among these molecules, FOXO1 is important as a mediator of stromal cell decidualization, protection against oxidative damage and menstrual shedding^25^. Like *LINC473*, FOXO1 is also an induced target of PKA pathway^26^. Transcriptome assay reveals that over half of the FOXO1 target genes were regulated under decidualization^26^. During decidualization, FOXO1 is required for the PGR binding to its targets. The comparison of FOXO1 and PGR ChIP-seq data reveals over 75% co-occupancy^26^. Decreased FOXO1 is related to decidualization failure in women with endometriosis^27^. MiRNA regulation on FOXO1 has been reported during human decidualization^28^. Our results extend the finding that FOXO1 is also regulated by LincRNA during decidualization.

In summary, we have shown that *LINC473* can significantly contribute to decidualization. However, the cellular function of LincRNAs remains enigmatic. In this regard, our data contribute to a growing body of literatures supporting the importance of LincRNA in cellular biology, especially during decidualization. As the potential of LincRNA to be a therapeutic target has been discussed recently^29^, the identification and functional analysis of *LINC473* may add another layer of mechanism and foster novel markers or therapeutic options for clinical application, such as infertility and reasonable contraception.

## Materials and Methods

### Cell culture and *in vitro* induced decidualization

Human uterine stromal cells were isolated as previously described^30^. Human endometrial samples were collected from normally cycling women undergoing hysterectomy or endometrial biopsy with written informed consent. All human procedures were approved by the Institutional Committee on the Use of Human Subjects in Medical Research of Bailu Hospital (Xiamen, China). The methods were carried out in accordance with the approved guidelines by South China Agricultural University. Briefly, endometrial tissues were minced into small pieces and incubated in DMEM/F-12 containing 0.2% type I collagenase (Gibco) for 60 min at 37 °C. Single cells were dispersed mechanically by vibrating. The resultant single cell suspension was separated by successive filtrations through a 200 μm cell strainer. To induce decidualization, cells were treated with 10 nM E2 (Sigma), 0.5 mM db-cAMP (Sigma) and 1 μM medroxyprogesterone acetate (Sigma).

### Sequence conservation analysis

Human *LINC473* sequence was retrieved from Ensembl (http://www.ensembl.org/). The conserved sequences in other species were identified by PhastCons, UCSC (http://genome.ucsc.edu/). When more than 90% of human *LINC473* was covered in the alignment, the segment was considered as present in other species. All identified sequences were aligned using ClustalW2 to construct the phylogenetic tree.

### Real-time RT-PCR

Cells were harvested with TRIzol Reagent (Sigma) and RNA was extracted according to the manufacturer’s instructions. After digesting with RQ1 deoxyribonuclease I (Promega), total RNAs were reverse transcribed into cDNA with PrimeScript reverse transcriptase reagent kit (TaKaRa) and then real-time RT-PCR was performed using SYBR Premix Ex Taq kit (TaKaRa) on the Rotor-Gene Q system (BioRad). All reactions were run in triplicate. The ΔΔCt method was employed to determine relative changes of gene expression compared to GAPDH. Primer sequences used were listed in Table S1. Quantifications were performed with standard curves generated with pGEM-T plasmids containing specific cDNA amplicons.

### Western blot

In brief, HESCs were lysed in lysis buffer (50 mM Tris-HCl, pH7.5, 150 mM NaCl, 1% Triton X-100, and 0.25% sodium deoxycholate). Protein concentration was quantified using the BCA kit (Applygen). Lysates were then resolved on a 10% SDS-PAGE gel and transferred onto PVDF membrane (Millipore). After blocking with 5% skim milk (Sangon), membranes were probed with the corresponding antibodies for phospho-STAT3 (Cell Signaling), total STAT3 (Cell Signaling), and Tubulin (Cell Signaling), respectively. Membranes were then incubated with the matched secondary antibodies conjugated with HRP (1:5000). Signals were detected with ECL kit (Pierce).

### Plasmid Construction and Transfection

Different lengths of *LINC473* promoter fragments were amplified using primers listed in Table S2 by PCR from human genomic DNA. The size of PCR products and their distance from TSS were also shown in Table S2. The amplified fragments were digested by restriction enzymes Mlu I and Hind III, and inserted into the upstream of the start codon of luciferase gene in the pGL3-basic vector (Promega).

The mutation of STAT3 binding site in the promoter of *LINC473* was generated with Quik ChangeTM Site-Directed Mutagenesis Kit (Stratagene, CA, USA). The primers used for the mutation were 5′-TCCGGCTGAACCCGCCCGAGCGCCCGCTC -3′and 5′-GAGCGGGCGCTCGGGCGGGTTCAGCCGGA -3′, in which the mutated sites were underlined. All the constructs were verified by sequencing.

### Promoter reporter assay

Transfections were performed with Lipofectamine 2000 (Invitrogen) according to the manufacturer. Six hours after reporter transfection, cells were treated with cAMP, H89 or S3I, respectively. Cell extracts were assayed for firefly and renilla luciferase activities (Promega E1910).

### siRNA transfection

RNA interference was performed by using synthetic siRNA duplexes. siRNA oligonucleotides targeting *LINC473* were designed and synthesized by Genepharma (Shanghai, China). HESCs were transfected with siRNA using Lipofectamine 2000 (Invitrogen) as manufacturer’s instructions. Cells were harvested using TRIzol (Invitrogen) for Real-time PCR or lysis buffer for Western blot assay.

## Additional Information

**How to cite this article**: Liang, X.-H. *et al.* Non-coding RNA *LINC00473* mediates decidualization of human endometrial stromal cells in response to cAMP signaling. *Sci. Rep.* **6**, 22744; doi: 10.1038/srep22744 (2016).

## Supplementary Material

Supplementary Information
