# Supplementary material for: Non-coding RNA LINC00473 mediates decidualization of human endometrial stromal cells in response to cAMP signaling
Source: Sci Rep. 2016 Mar 7;6:22744. doi: 10.1038/srep22744 (PMC4780002; doi:10.1038/srep22744)

# **Non-coding RNA *LINC473* mediates decidualization of human endometrial stromal cells in response to cAMP signaling**

Xiao-Huan Liang, Wen-Bo Deng, Yue-Fang Liu, Yu-Xiang Liang, Zong-Min Fan, Xiao-Wei Gu, Ji-Long Liu, Ai-Guo Sha, Hong-Lu Diao, Zeng-Ming Yang

**Supplemental Table 1 Primers used in this study**

| Primer Name | Sequences (5'-3') | Product Size (bp) | Genbank Number |
|---|---|---|---|
| *LINC473* | GATGGAAAGGAGGGAAGG | 200 | NR_026860 |
| | CACAGTGGGTCCAGGGTT | | |
| GAPDH | GAAGGTGAAGGTCGGAGT | 94 | BC023632 |
| | GATGGCAACAATATCCACTT | | |
| STAT3 | CACTGTATCAGCATAGCCTTTC | 115 | NM_139276 |
| | GGTTTCACCGTGTTAGCC | | |
| IL-11 | AAGCTGCAAGGTCAAGATG | 122 | NM_001267718 |
| | GCTGGGTGGCGTTCTATC | | |
| PRL | AAGCTGTAGAGATTGAGGAGCAAA | 76 | NM_000948 |
| | TCAGGATGAACCTGGCTGACTA | | |
| IGFBP1 | CCAAACTGCAACAAGAATG | 87 | NM_001013029 |
| | GTAGACGCACCAGCAGAG | | |
| FOXO1 | CGAGCTGCCAAGAAGAAA | 105 | NM_002015 |
| | TTCGAGGGCGAAATGTAC | | |
| PGR | TGCCTATCCTGCCTCTCAATCAC | 78 | NM_000926.4 |
| | CGCCGTCGTAACTTTCGTCTTC | | |
| CEBPB | AACTCTCTGCTTCTCCCTCTG | 113 | NM_001285878.1 |
| | TGCGTCAGTCCCGTGTAC | | |
| BMP2 | TGGAAGGTTACTCTGGCAAAG | 79 | NM_001200.3 |
| | CTGGACTTGAACTTGTGAACTC | | |
| WNT4 | CATGCAACAAGACGTCCAAG | 121 | NM_030761.4 |
| | AAGCAGCACCAGTGGAATTT | | |
| HOXA10 | GGTCTTACATTGCCTGACTAA | 172 | NM_018951.3 |
| | AGATAGGGAGAATTGTGGTGT | | |

| | | | |
|---|---|---|---|
| HOXA11 | CCCATTGAATCTCCTTTGC<br>CAGTTGCCTGTATAAGTGCTG | 130 | NM_005523.5 |

**Supplemental Table 2 Primers used for promoter assay**

| Primer Name | Sequences (5'-3') | Location (bp) |
|---|---|---|
| LINC473-1331-F: | CGAGCTCTTACGCGTTTTATGAAGCTGCG CCCTGTGCCTG | -1246 bp |
| LINC473-808-F: | CGAGCTCTTACGCGTAGGCGAGGGCGCCT GTCCCCACGCG | -718 bp |
| LINC473-553-F: | CGAGCTCTTACGCGTGGGTCGGGTTGGGG GGACTGCGGGG | -468 bp |
| LINC473-249-F: | CGAGCTCTTACGCGTACTGTTCTGGCAAC GAGGCTACAGA | -163 bp |
| LINC473-135-F: | CGAGCTCTTACGCGTGTCGTTCCTGAGAG CACAAGCACCC | -50 bp |
| LINC473-R: | CGGAATGCCAAGCTTGCAACTTCGGACTC AGACCT | |
| LINC473-mut-F: | TCCGGCTGAACCCGCCCGAGCGCCCGCTC | |
| LINC473-mut-R: | GAGCGGGCGCTCGGGCGGGTTCAGCCGG A | |

**Supplemental Figure 1** PGE2 regulation on *LINC473*, IL-11 and STAT3. (A) PGE2 up-regulates *LINC473* RNA expression in a dosage-dependent manner. (B) Time course for PGE2 treatment. (C) IL-11 mRNA is induced by PGE2. (D) PGE2 regulation on phosphorylated STAT3 in HESCs. (E) H89 impedes the PGE2 induction on *LINC473*. Each treatment was performed with at least three biological replicates. Error bars represent standard errors. *, $P < 0.05$.

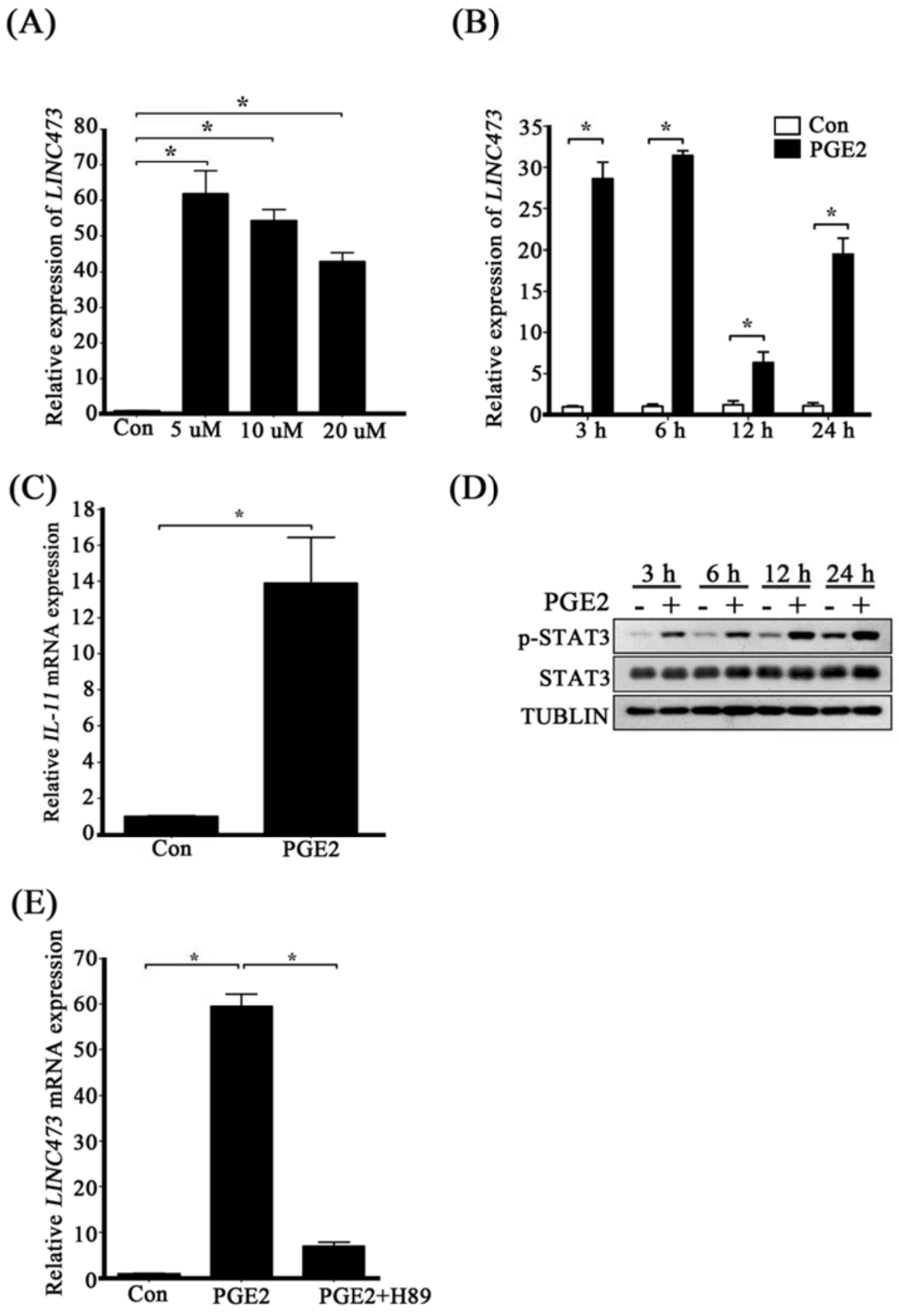

Supplement: Supplementary Information [file srep22744-s1.pdf]
